# Utilization of Silica Filler as Reinforcement Material of Polylactic Acid (PLA) in 3D Printing Applications: Thermal, Rheological, and Mechanical Performance

**DOI:** 10.3390/polym16101326

**Published:** 2024-05-08

**Authors:** Vasileios Stratiotou Efstratiadis, Apostolos Argyros, Pavlos Efthymiopoulos, Georgios Maliaris, Nektarios K. Nasikas, Nikolaos Michailidis

**Affiliations:** 1 Physical Metallurgy Laboratory, Mechanical Engineering Department, School of Engineering, Aristotle University of Thessaloniki, 54124 Thessaloniki, Greece; vstratio@auth.gr (V.S.E.); aargyros@auth.gr (A.A.); 2 Centre for Research & Development of Advanced Materials (CERDAM), Aristotle University of Thessaloniki and Texas A&M Engineering Experiment Station, Centre for Interdisciplinary Research and Innovation, 57001 Thessaloniki, Greece; 3 Additive Manufacturing Laboratory, Department of Chemistry, International Hellenic University, 65404 Kavala, Greece; pefthymi@chem.ihu.gr (P.E.); gmaliari@chem.ihu.gr (G.M.); 4 Department of Military Sciences, Division of Mathematics and Engineering Sciences, Hellenic Army Academy, 16673 Vari, Greece; nasikas@sse.gr

**Keywords:** sustainability, recycle, additive manufacturing, waste recycle, glass beads, glass recycle, composite materials

## Abstract

Glass was introduced as an additive to filaments used for the manufacturing of composite materials, employed by Additive Manufacturing applications. Glass accounts for a large waste electric and electronic equipment (WEEE) percentage, and its recovery and recycling can lead to the production of sustainable composite materials. In this work, poly(lactic acid) (PLA)/commercially available silicon oxide composite filaments were manufactured and their structural, thermal, rheological, and mechanical properties were assessed. Scanning Electron Microscopy confirmed the 1:2 ratio of silicon: oxygen, along with the relatively low adhesion between the filler and the matrix. Differential Scanning Calorimetry presented steady glass transition and melting temperatures of composites, whereas a crystallization temperature of 10% wt. and a crystallinity of 15% wt. composite slightly increased. Rheological analysis showcased that the viscosity of the composite filaments decreased compared to PLA (10–100 compared to 300–400 Pa·s), with a more shear-thinning behavior. Dynamic mechanical analysis exhibited increased elastic, flexural moduli, and flexural strength of composites (up to 16, 23, and 11%, respectively), whereas tensile strength and elongation decreased. The affordability of raw materials (with the future introduction of recycled ones) and the minimal processing steps can lead to the potential scaling up of the study.

## 1. Introduction

A significant amount of waste electrical and electronic equipment (WEEE) is produced globally on an annual basis, especially in developed countries, fueled by the rapid economic expansion and technological advancements in the electrical and electronic equipment sector [1].

The European Union (EU) updated the WEEE guidelines in 2012, including photovoltaic (PV) components among the discarded electronic devices in the WEEE categories [2]. PV cells are produced by applying one or more layers of photosensitive semiconductor compounds onto a non-conductive support substrate, such as glass, which constitutes 85% of the solar panel [3,4].

The collection and recycling of glass from WEEE present the potential of shifting towards a minimum waste supply chain, prioritizing sustainability, and emphasizing circular economy principles.

Silica glass can be found in the packaging industry in every form, in End-of-Life WEEE (in PV in the form of infrastructure for the collection, recycling, and safe disposal of WEEE) that will act as recycled raw materials (RM). Additionally, ongoing data collection and analysis will enable authorities to monitor progress, identify emerging trends, and adapt strategies accordingly. Furthermore, there is a pressing need for heightened public awareness campaigns for substrates or in the form of fibers in Printed Circuit Boards (PCB). Furthermore, it is one of the most widely recycled products, making it an excellent candidate as a filler in thermoplastic filaments, aiming to enhance the inherent properties of the final product [5]. Recent data show that the collection and recycling of glass packages reached about 80% in Europe in 2019, and a total of almost 3.5 million tons of recycled glass is used in the US in various applications [6,7].

The burgeoning issue of WEEE within the EU demands immediate attention from authorities. As the production and consumption of electronic devices escalate, so does the accumulation of WEEE, posing significant environmental and health hazards. To mitigate these risks, authorities must take proactive measures to address the management and disposal of WEEE, reaching targeted checkpoints [8]. It is imperative for policymakers to enact stringent regulations and establish robust encourage responsible disposal practices among consumers [8].

Material Extrusion three-dimensional (MEX 3D) printing is a type of Additive Manufacturing process, where a thermoplastic filament is heated and extruded through a nozzle, layer by layer, to build up a 3D object based on a computer-aided design (CAD) model [9]. Advantages of MEX 3D printing include cost-effectiveness, versatility of thermoplastic materials, speed, and ease of use [10,11].

MEX 3D printing allows for the creation of complex geometries that would be difficult or impossible to achieve with traditional manufacturing methods, as the layer-by-layer deposition of material enables the fabrication of structures with intricate details [10]. It offers flexibility to incorporate various materials (thermoplastic materials and additives such as fibers or fillers) into the printing process, allowing for the creation of functional components with diverse properties [12]. Finally, whether it is adjusting the size, shape, or features of a component, MEX allows for rapid prototyping and iteration, enabling quick testing and refining of designs before final production [13].

Currently, poly(lactic acid) (PLA) is one of the main commercially available thermoplastics for MEX 3D printing [10]. It is bioabsorbable, biocompatible and recyclable. Applications of 3D-printed PLA parts to medical, textile, and other industries are widespread, as PLA degradation products are found to be non-polluting and non-toxic [14,15]. Furthermore, lactic acid is a naturally occurring organic compound [16].

Nevertheless, PLA has low toughness and high brittleness, mainly due to its relatively low glass transition temperature (T_g_). T_g_ of amorphous PLA is 55–65 °C and is a function of the PLA molecular weight and stereochemistry. PLA’s cold crystallization temperature (T_cc_) lies between 80 and 150 °C, and melting temperature (T_m_) between 150 and 160 °C, respectively [15,17,18]. Above T_g_, amorphous PLA transitions from glassy to rubbery and behaves as a viscous fluid upon further heating. Below T_g_, it acts as a brittle polymer [16]. PLA composites can be used to enhance the physicochemical and mechanical properties of thermoplastics, as the main challenge in manufacturing parts with the MEX process is to overcome their low mechanical properties [11,15].

When enhanced mechanical properties are sought, semi-crystalline PLA is favored over amorphous polymers. It demonstrates an estimated tensile strength within the range of 50–70 MPa, a flexural modulus of 3–4 GPa, flexural strength of approximately 90 MPa, and an elongation of 5% [19,20,21].

The characteristics of composite materials, both physical and mechanical, are heavily influenced by the distribution of fillers within polymer matrices. When fillers are uniformly dispersed, the resulting properties are typically improved. Conversely, inadequate dispersion can lead to a significant decline in these properties. [22]. The filler morphology, size, and content are factors of paramount importance as they can lead to the blockage of the printer’s nozzle [23,24]. In the plastics industry, short fibers, (e.g., glass fibers) are mainly used for enhancing mechanical properties of MEX 3D-printed composites [25]. In order to ensure that a brittle, rigid filler will not deteriorate, the properties of composites, impact modifiers, plasticizers, and silanes are often used. Impact modifiers enhance the toughness and resilience of PLA-based composites as they disperse within the matrix and absorb energy during mechanical stress [26]. Some of the most widely used for PLA are poly(ethylene terephthalate) and poly(vinyl alcohol) fibers [26]. Plasticizers improve the flexibility and processability of polymers, as they increase the mobility of polymer chains, thereby reducing T_g_ and enhancing flexibility [27]. Examples are citrate esters (triacetine citrate, tributyl citrate, and triethyl citrate) [28]. Silanes are organo-coupling agents that contain functional groups, which can create siloxane bonds between organic polymer matrices and inorganic fillers, such as GBs, and increase interfacial adhesion (such as hexadecyltrimethoxysilane and tetraethyloxysilane) [29,30].

The primary scope of this research is to manufacture MEX 3D-printed composite materials, by incorporating commercially available Glass Beads (GBs) as filler in the PLA matrix. The composites’ properties will be evaluated in comparison to pure PLA MEX 3D-printed products through various analyses, including Scanning Electron Microscopy (SEM), Differential Scanning Calorimetry (DSC), rheological analysis, and dynamic mechanical analysis [31]. The focus of this study is the investigation of the behavior of commercially available GBs as fillers in the PLA matrix without any additional modifications. This decision stemmed from the objective of understanding the intrinsic properties and potential feasibility of using pristine GBs as fillers in PLA composites and isolating their effect on the properties of composites, laying the groundwork for further investigation. Moreover, this study serves as the initial stage for upscaling, with the intention of eventually incorporating recycled glass from WEEE as fillers in PLA and developing sustainable and scalable solutions for composite materials.

## 2. Materials and Methods

**Figure 1:**
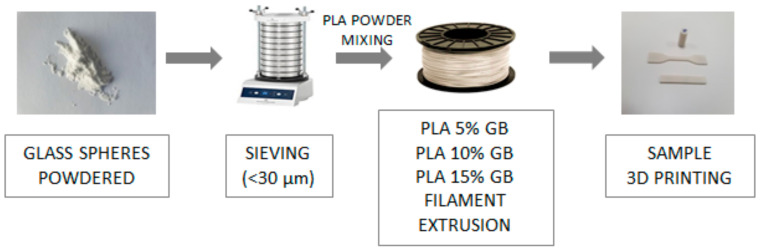
Process followed for the printing of composites.

The matrix material for this study was the extrusion grade PLA Ingeo™ Biopolymer 4043 D designed for monofilament MEX, by NatureWorks LLC (Plymouth, MN, USA). The material, obtained in powder form, was procured from 3devo B.V. (Utrecht, The Netherlands), with a density of 1.24 g/cm^3^ according to ASTM D792 [32], as indicated in the manufacturer’s technical data sheet. Additionally, SiO_2_ spherical microparticles, with a diameter of 9–13 μm and a density of 1.1 g/cm^3^, were acquired from Sigma-Aldrich (St. Louis, MO, USA). Before mixing it with PLA powder, the SiO_2_ powder underwent sieving to ensure dimensional uniformity, as illustrated in Figure 1.

Three composite filaments with 5, 10, and 15% wt. filler loadings were denoted as PLA_GB_f, with f being the wt% of the filler, namely 0.05, 0.1, and 0.15, respectively. Drying of both materials was carried out at 85 °C for 4.5 h in a forced convection oven. Each pre-weighted PLA and filler powder mixture was homogenized using a rotating cylindrical drum. Subsequently, each blend was introduced into the hopper of a Next 1.0 Advanced desktop single-screw filament extruder, manufactured by 3devo B.V in Utrecht, The Netherlands. This extruder is outfitted with a self-regulating system for controlling the filament diameter, which was calibrated to 1.75 mm. Throughout the extrusion process, the screw speed remained consistently set at 3.5 rpm. The temperature profiles of the extruder’s heating zones that were employed were 170, 185, 190, and 170 °C, respectively, upon moving from the hopper to the nozzle, as recommended by its manufacturer for PLA monofilament extrusion. Crystal formation occurs between T_g_ and T_m_ of the polymer during cooling, as it is heated above its T_m_ [33].

Finally, all filaments were placed in a Prusa i3 MK3S, for the final composites to be 3D printed and tested [31]. Printing conditions are given in Table A1 in Appendix A.

Fillers, composite filaments, and final products were analyzed by SEM to provide information about their morphological structure, their elemental analysis (using an integrated energy-dispersive X-ray diffraction (EDX) detector), the structure of crystalline formations, and dispersity. The analysis was carried out by Phenom ProX G6 by Thermofisher Scientific (Waltham, MA, USA), with electron-optical magnification upper limit of up to 350,000 times [34,35]. The specimens were mounted on a sample holder with carbon tape, using gold coating.

DSC enables the measurement of thermal events such as glass transition, crystallization, melting, oxidation, enthalpies, and other thermal events [36]. By extending the analysis of heat flow measurements, heat capacity, compatibility, and stability of sample blends, the effects of additives on crystallization can be obtained. The principle behind DSC involves comparing the heat flow of the sample and a reference material. During a change of phase or a transformation of the sample, any energy difference between the sample material and the reference is measured, providing valuable information about its thermal properties and behavior. The sample is enclosed in an aluminum pan, next to an empty reference one [37]. DSC analysis of the fillers (in the same temperature range as the filaments), pure PLA, and composite filaments was conducted using a Discovery DSC 25 instrument from TA Instruments (New Castle, DE, USA) equipped with a Cooling System (temperature range between −90 and 550 °C and heating rates 0.01–100 °C/min) [36]. In the present study, DSC analysis had an initial heating stage of 10 °C/min to 230 °C, followed by a cooling stage of 10 °C/min to 25 °C and a 2nd heating stage of 10 °C/min to 230 °C to eliminate the influence of the polymer’s thermal history during production. The analysis took place under nitrogen atmosphere and the mass of the samples ranged from 6 to 6.8 mg.

Rheological analysis was conducted on all filaments. Rheometry, a technique that assesses the extent of deformation exhibited by a material or liquid under the influence of applied force, was employed. The study of material behavior, specifically viscosity, under shear stress and strain, serves as the foundation for rheology. The assessments were carried out using a Discovery Hybrid Rheometer, HR20, provided by TA Instruments (New Castle, DE, USA), featuring a parallel plate configuration, and a heating system (temperature range between 160 and 600 °C). The incorporation of cutting-edge measurement technology enhances sensitivity and precision, allowing for the measurement of lower viscosities with reduced material consumption [38]. The experiments took place at 215 °C (same as the nozzle temperature for the printing of the first layer), the torque range was 1–25,000 μN·m, and the gap between the plates was 1 mm.

Ultimately, the assessment of mechanical performance, including tensile strength and 3-point bending, for both pure PLA and composite filaments was conducted utilizing the Electroforce 3550, from TA Instruments (Eden Prairie, MN, USA). This system boasts a dynamic force capability of 15 kN, employing two linear motors in a synchronized manner. Specifically, it utilizes various force and frame configurations, enabling precise control of force, displacement, or strain within a broad frequency range during tensile or flexural tests. Moreover, the Electroforce 3550 facilitates dynamic characterization tests for a diverse range of materials, components, and devices [39]. The speed used in the present study was 2 mm/min.

## 3. Results

### 3.1. SEM Analysis

**Figure 2:**
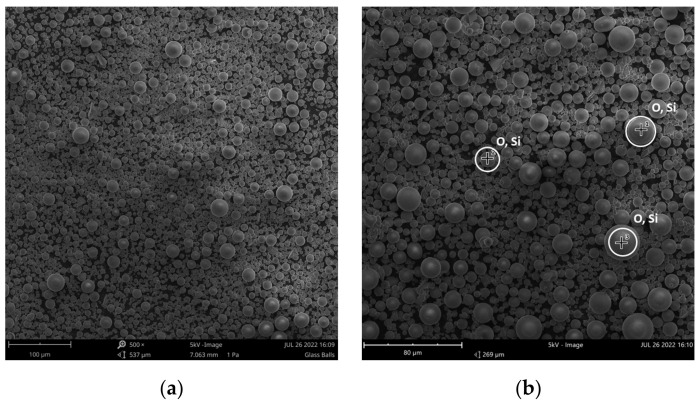
SEM analysis of fillers at 500× (**a**) and 1000× (**b**) magnification and point selection EDX analysis.

**Figure 3:**
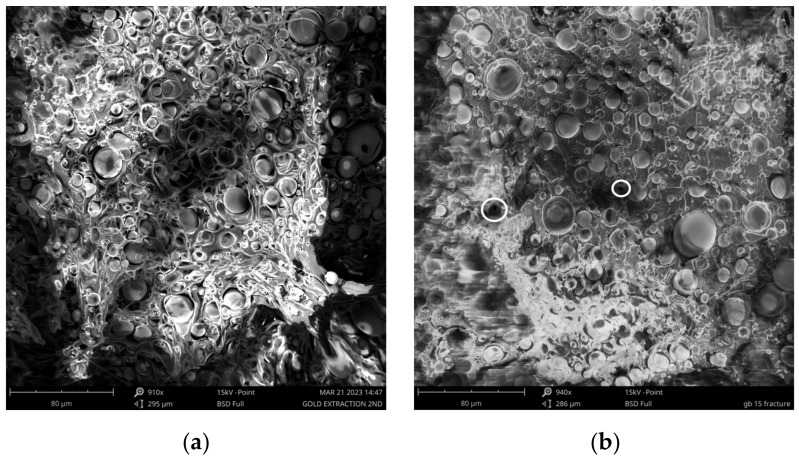
SEM analysis of PLA_GB_0.15 composite filament (**a**) and fracture surface of 3D-printed composite sample (**b**).

GBs are visible in Figure 2a and the element identification tool during SEM analysis in three different points (Figure 2b) presents their composition in Table 1. SEM analysis of PLA_GB_0.15 filament is given in Figure 3a, where the spherical filler particles in the polymeric matrix are clearly visible. Furthermore, Figure 3b shows the fracture surface of the PLA_GB_0.15 composite 3D-printed sample. There are some spherical voids on the fracture surface of the PLA matrix, indicating the presence of pulled-out GBs. The beads are not fractured. Interfacial adhesion between the filler and PLA matrix may not be strong enough to withstand the applied stress, leading to the debonding of the filler from the matrix leaving behind cavities, prior to a possible ductile deformation of the matrix, leading to brittle failure [40]. Although there is a uniform dispersion of the filler in the matrix, there are also clusters, leading to the deterioration of stress transfer.

All points present a similar composition of silicon (Si) and oxygen (O), confirming the nature of the filler (SiO_2_).

### 3.2. DSC Analysis

**Figure 4:**
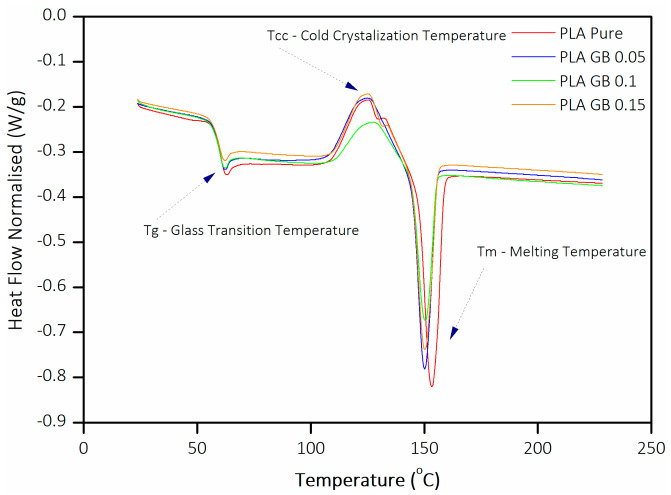
DSC analysis of pure PLA and composite filaments.

The analysis shown in Figure 4 reveals that the transition temperature from a hard, brittle state to a rubbery/viscous state, namely T_g_, of composites is marginally lower compared to PLA. In particular, T_g_ decreases with increasing filler loading. Following the same trend, composite T_m_ is decreased compared to PLA and decreases with increasing GB content.

All samples present an exothermic cold crystallization peak around 110 °C. T_cc_ of pure PLA, PLA_GB_0.05, and PLA_GB_0.15 are similar, whereas the crystallization range of 10% additive concentration composite (PLA_GB_0.1) is broadened and displaced to a slightly higher temperature as seen in Table 2. There is more than one structure of crystals in the composites (α′ and α) [41]. Upon heating, PLA initially transitions from the glassy state to the rubbery state, where molecular mobility increases [41,42]. As the temperature further increases, the polymer chains rearrange into the α′-crystalline form, leading to a slight displacement of the curve [29,43,44]. The α′-crystalline form of PLA is less ordered and exhibits lower crystallinity levels than the α-crystalline form.

**Figure A1:**
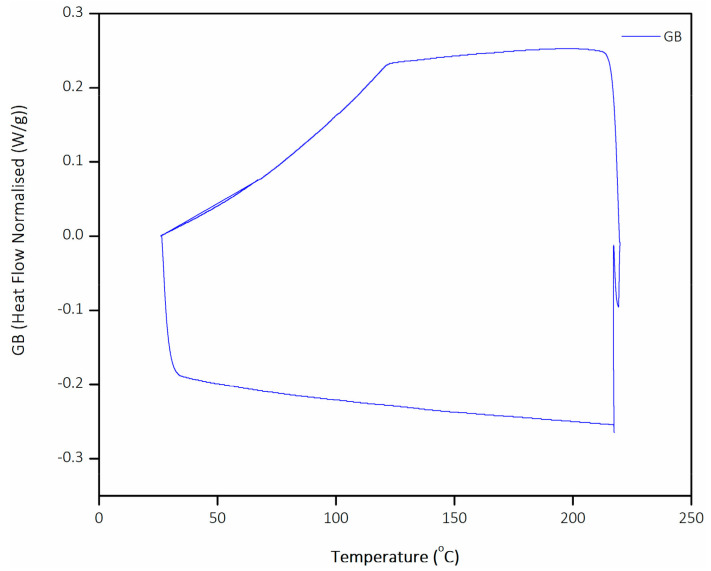
GB DSC analysis.

T_cc_ is also influenced by crystallinity (X_c_), as seen in Table 3 [45]. However, the addition of fillers did not influence T_g_, T_cc_, and T_m_ significantly. The repeatability (three experiments per sample), the low scattering of the values, and the lack of polymer decomposition are in accordance with the results from Malinowski et al. [46]. DSC analysis of GBs is given in Figure A1 in Appendix A and shows no thermal transitions in the range of 100–150 °C [47].

X_c_ of composites filaments was calculated using Equation (1) [48]

(1) $$X_{c}\left(\%\right)=\frac{{\Delta H}_{m}-{\Delta H}_{\mathrm{cc}}}{{\Delta H}_{0}\varphi }100$$

where ΔH_m_ is the melting enthalpy and ΔH_cc_ is the cold crystallization enthalpy, respectively (integral of the area of endothermic and exothermic peaks), φ is the PLA percentage in the composite and ΔH_0_ is the melting enthalpy of 100% crystalline PLA (93 J/g), according to the literature [49]. X_c_ of pure PLA and composite filaments is shown in Table 3.

X_c_ is initially decreased. However, the addition of a higher filler percentage leads to its increase. The addition of 15 wt% filler leads to a slight increase in X_c_ compared to pure PLA. As seen in Figure 4, PLA_GB_0.05 and 0.1 do not exhibit overlapping exothermic peaks, but PLA-GB_0.15 did, meaning that there was a phase transition from α′ to α-crystalline form, hence the slightly increased crystallinity.

GBs, being pristine inorganic particles, without any modifications, most likely do not participate in the crystallization process of PLA. They act as fillers, dispersed within the polymer matrix, without undergoing any significant structural changes or exhibiting nucleating capabilities for PLA. As a result, they do not influence the formation of PLA crystals or alter the crystallization kinetics [41,42].

These results indicate that GBs in large concentrations did not increase crystallinity significantly. This is supported by the SEM figures, where it is concluded that there is no good interfacial adhesion. The initial decreasing trend of X_c_ can be attributed to micro-SiO_2_ particles hindering the movement of the PLA molecular chains; hence, the chains available for crystallization are reduced.

### 3.3. Rheological Analysis

**Figure 5:**
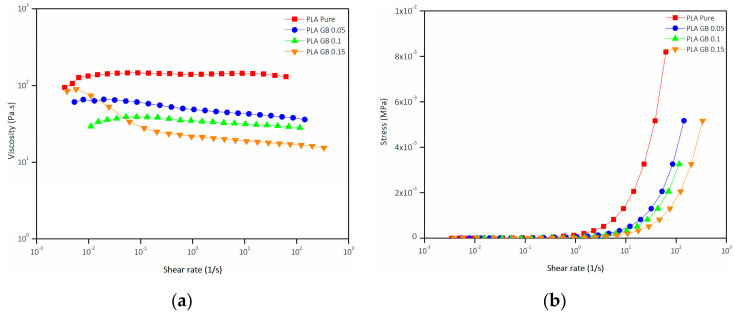
Viscosity against shear rate (**a**), stress against shear rate (**b**) for pure PLA and composite filaments.

Figure 5a,b display the logarithmic curve of viscosity and nominal curve of stress respectively, as a function of the logarithm of shear rate for pure and composite filaments. It is important to highlight that three separate experiments were conducted for each sample to validate the results and minimize any potential errors.

PLA exhibits a steady viscosity (with the exception of the initial shear rate increase) with an increasing shear rate of up to 0.1 rad/s. As the shear rate increases beyond that point, viscosity decreases, and the sample exhibits pseudoplastic behavior. The viscosity of the composites follows the same trend, exhibiting pseudoplastic behavior [50,51].

Composite filaments showcase lower viscosity than pure PLA filament, throughout the tested shear rate range, as GBs increase the lubrication effect on the PLA chains due to their spherical shape. As the percentage of hard GBs increases, it leads to packed particles [52]. As a result, the slippage between the filler and the PLA particles is facilitated and the polymer chain strength is weakened against the imposed shear stress [53,54]. Moreover, shear stress tends to orient the additives in bands, resulting in more shear-thinning behavior. Finally, the viscosity of PLA_GB_0.05 is slightly higher than PLA_GB_0.1, meaning that higher additive concentration enhances the slippage phenomena, lowering the viscosity and resulting in stronger shear-thinning behavior. This is also confirmed by the case of PLA_GB_0.15, where its initial viscosity is increased compared to the composite filaments, but it also presents the strongest shear-thinning behavior, resulting in the lowest viscosity of all samples occurring in larger shear rates.

### 3.4. Dynamic Mechanical Analysis—Tensile and 3-Point Bending Tests

**Figure 6:**
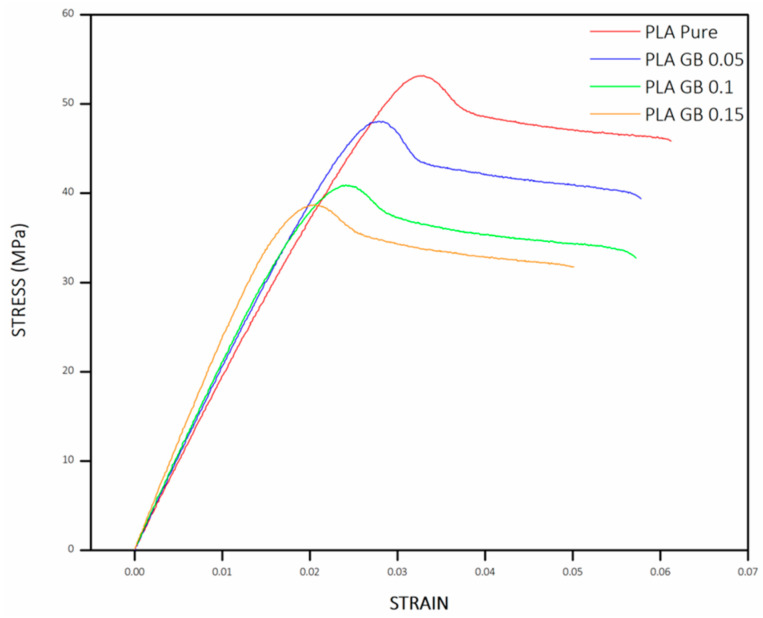
Stress/strain diagram of all 3D-printed products—tensile tests.

**Figure A2:**
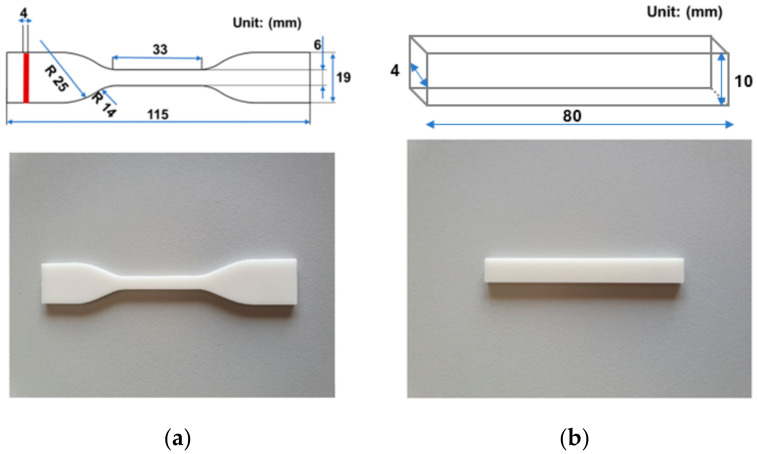
D638-10 TYPE IV ASTM Dimensions (Reprinted with permission from [66], 2024, Springer Nature BV) (**a**) and ISO 178 Parallelepiped Dimensions (**b**).

Three experiments were carried out for every sample to minimize potential errors. For every test, samples were printed, following ASTM D638-10 TYPE IV [55] dimension standards for tensile and ISO 178 [56] for 3-point bending tests, respectively (standards dimensions are given in Figure A2a,b and sample dimensions are given in Table A2 in Appendix A, respectively). The stress/strain graph obtained from the tensile tests, as depicted in Figure 6, allows for the calculation of the elastic (or Young’s) modulus E, which is determined from the gradient of the linear segment of the graph.

**Figure 7:**
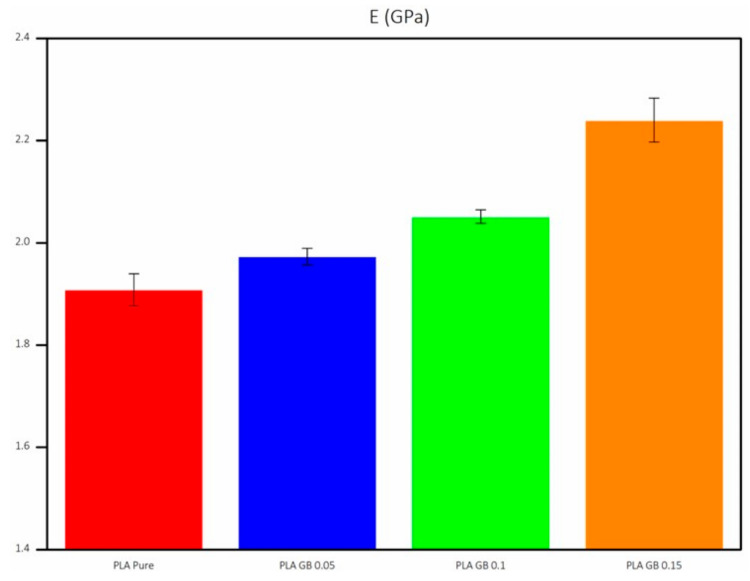
Elastic modulus of all 3D-printed products.

Figure 7 presents the average E for all final products, along with the statistical error from the multiple experiments. It is observed that incorporating fillers results in higher E and stiffness, which is in accordance with the literature [57,58]. This implies that more stress is required for the sample to undergo the same displacement within the elastic range, hence the higher slope. The addition of fillers, with higher rigidity and increased crystallinity (notably in the PLA_GB_0.15 composite), contributes to stiffening the polymeric matrix [57,58]. Consequently, the composite displays greater resistance to deformation compared to pure PLA.

The reinforcement mechanism is attributed to the transfer of stress from the matrix to GBs via a shear transfer mechanism, owing to the tendency of hard fillers to concentrate stresses. Tensile strength denotes the peak strength achieved during testing, just before the sample fractures, and it is quantified using Equation (2):

(2) $$\sigma =\frac{F}{A}$$

where F is the tensile force, (N), and A is the nominal cross-section of the specimen.

**Figure 8:**
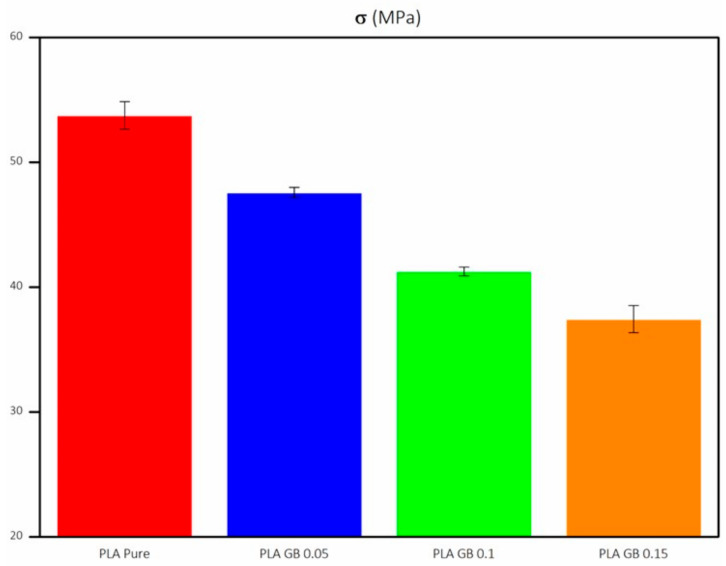
Tensile strength of all 3D-printed products.

Tensile strength (σ) decreases with the increasing filler percentage and decreasing PLA percentage. σ of composite filaments is lower than σ of 95 and 90 wt% of pure PLA, respectively (Figure 8). Although there is a uniform dispersion of the filler in the matrix as seen in Figure 3b, there are filler clusters, which can compromise the transfer of stress between the matrix and GBs, leading to the deterioration of tensile strength. This occurrence disrupts the matrix’s continuity and constrains the polymer chains’ capability to endure stress [29,57,58].

Finally, Equation (3) is used for the calculation of the elongation up to the breaking point of the sample is calculated using Equation (3):

(3) $$\varepsilon =\frac{l-l_{0}}{l_{0}}100$$

where l = final normalized gage length, (mm), and l_0_ = initial gage length.

**Figure 9:**
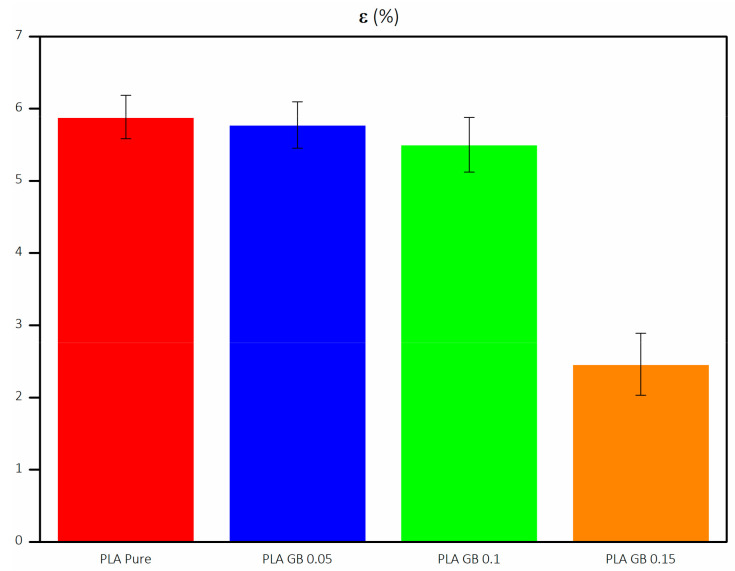
Elongation of all 3D-printed products.

ε decreases as PLA % decreases and GBs increase. GBs are rigid fillers, which present significantly lower ductility than PLA. As a result, the higher filler concentration decreases the deformation of the polymer (Figure 9). Specifically, PLA_GB_0.15 presents significantly decreased ε than the rest of the composites, as GBs may have substituted structural sections of the polymeric matrix. Furthermore, the addition of silicon oxide can favor elongation up to an optimized concentration (ε of PLA_GB_0.05 and PLA_GB_0.1 are higher than that of 95 and 90% of pure PLA, respectively), after said optimized concentration, it leads to its decrease [59]. ε decreased with increased crystallinity, according to Yang et al. [60], and this is evident in the PLA_GB_0.15 sample. The mean results are given in Table 4.

**Figure 10:**
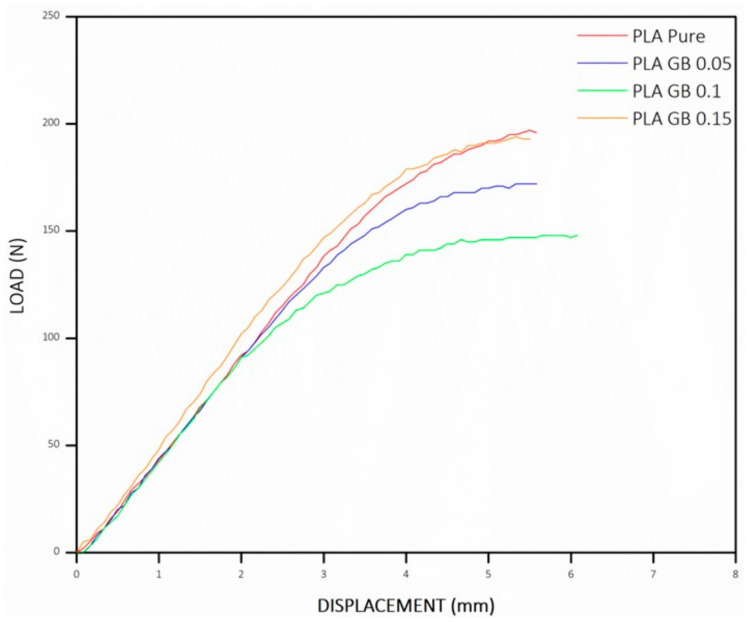
Load/displacement diagram of all 3D-printed products—3-point bending tests.

The load/displacement graph from the 3-point bending tests, as displayed in Figure 10, allows for the calculation of the flexural modulus (E_f_) and the flexural stress (σ_f_) using Formulas (4) and (5), respectively, with its maximum value observed just before the sample fractures.

(4) $$E_{f}=\frac{L^{3}m}{4{\mathrm{bd}}^{3}}$$

(5) $$\sigma_{f}=\frac{3\mathrm{FL}}{2{\mathrm{bd}}^{2}}$$

where L = support span, fixed at 60 mm, m = slope of the straight-line portion of the load/displacement graph, (N/mm), b = width of sample, (mm), d = thickness of sample, (mm), and F = load at a given point on the load/displacement graph, (N).

**Figure 11:**
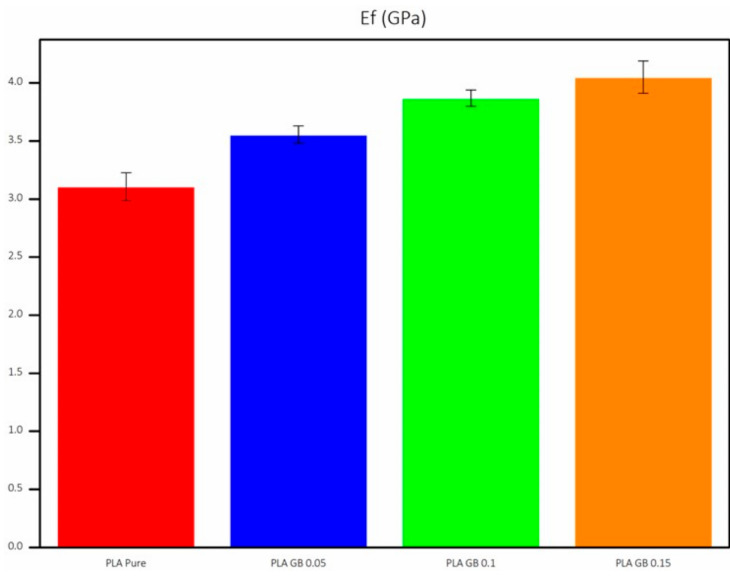
Flexural elastic modulus of all 3D-printed products.

Figure 11 presents the average E_f_ for all final products, along with the statistical error, following a similar trend to the tensile tests, as composites show an increased E_f_, becoming more resistant to deformation. A filler percentage increase leads to an increased E_f_.

**Figure 12:**
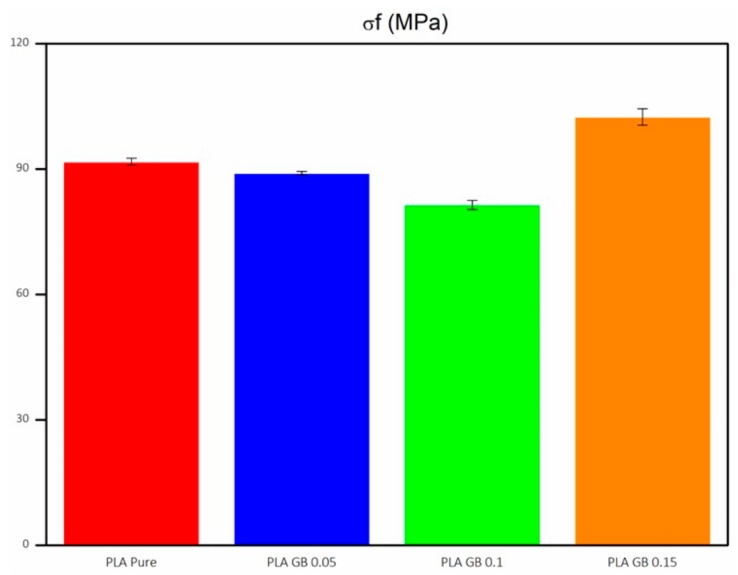
Flexural stress of all 3D-printed products.

The σ_f_ of composites is given in Figure 12 and follows the same trend as crystallinity. It decreases initially, as the filler percentage increases, and then increases after the addition of a filler percentage above 10%, with the maximum σ_f_ achieved at 15% filler concentration. Crystalline filler dispersion can influence flexural moduli and stresses [61,62]. Results are presented in Table 5.

## 4. Discussion

DSC analysis showed that T_g_, T_m_ of PLA composites in general were not influenced by the addition of GBs, whereas T_cc_ was mostly affected by the addition of 10% filler—less exothermic and slightly increased. Crystallinity slightly increased in the case of 15% wt. filler composite. Rheological analysis showed that composite filaments had lower viscosity than pure PLA, as GBs increased the lubrication effect, enhancing slippage phenomena between GB and PLA macromolecules. Lower melt viscosity can lead to a lower number of voids in the structure of the composite, but also to higher flowability; therefore, it can be challenging to extrude the composite filament with a uniform diameter [63,64].

Finally, dynamic mechanical analysis showcased that PLA_GB composites presented higher E and Ε_f_ than pure PLA, due to stiffness increase, as rigid structured fillers were added. As a result, the composite became more resistant to deformation in the elastic range. The σ, σ_f_, and ε of PLA_GB composites decreased compared to pure PLA, as PLA percentage decreased. The results between tensile and 3-point bending tests followed the same trend among samples. The exception of PLA_GB_0.15, which presented an increased σ_f_, compared to pure PLA and the composites is due to the increased crystallinity of the composite, as crystallinity can influence flexural stress.

## 5. Conclusions

Glass represents a predominant component within WEEE and commercial waste, and its recycling and reusability can lead to added-value materials, aligning with a sustainable design approach. This research investigated the effect of incorporating 5, 10, and 15% wt. of commercially available Glass Bead (GB) filler into PLA matrix on the rheological, thermal, and mechanical properties of the resulting composite 3D-printed products. This approach aimed to assess the feasibility of utilizing pristine glass into composites, enhancing their properties, and constituting the first stage of future studies, where recycled glass sourced from WEEE will be investigated and compared to pristine glass filler, promoting sustainability, and zero-waste policies, while offering tangible solutions for industries seeking to minimize their ecological footprint.

With their specific properties, PLA and GB composites are suitable for manufacturing functional parts in electronics, including casings, brackets, and connectors. The high precision achievable through 3D printing also makes them ideal for intricate electronic components. The lightweight nature of PLA and the ease of composite manufacturing enable the quick and cost-effective production of prototypes and molds for various industries such as automotive, aerospace, and consumer goods. Finally, the biocompatibility of PLA combined with the increased tensile strength provided by GBs makes these composites suitable for medical applications.

Future research will also explore composite filaments with higher glass powder concentrations and the incorporation of plasticizers and silanes to improve compatibility between the filler and the polymer matrix, due to the enhancement of their interfacial chemical affinity [65]. Additionally, the investigation of various recycled materials from WEEE, including critical metals and rare earth elements, as fillers in PLA and other polymer matrices such as acrylonitrile butadiene styrene (ABS), holds promise for further advancement.

## Figures and Tables

**Table 1:** EDX analysis of selected points.

Point	Element Number	Element Symbol	Atomic Concentration
1	8	O	79.57
	14	Si	20.43
2	8	O	79.44
	14	Si	20.56
3	8	O	76.50
	14	Si	23.50

**Table 2:** T_g_, T_cc_, and T_m_ of pure PLA and composite filaments.

Sample	T_g_ (°C)	T_cc_ (°C)	T_m_ (°C)
PLA	63.11	125.19	153.51
PLA GB 0.05	62.44	124.89	150.45
PLA GB 0.1	62.27	127.17	150.35
PLA GB 0.15	62.2	125.58	150.13

**Table 3:** X_c_ of pure PLA and composite filaments.

Sample	ΔH_m_ (J/g)	ΔH_cc_ (J/g)	φ	X_c_ (%)
PLA	17.45	−14.9	1	2.74
PLA GB 0.05	15.52	−13.51	0.95	2.27
PLA GB 0.1	12.36	−10.27	0.9	2.50
PLA GB 0.15	15.63	−13.26	0.85	3

**Table 4:** Mean values of E, σ, ε of pure PLA and composite 3D-printed samples—tensile tests.

Sample	E (GPa)	σ (MPa)	ε (%)
PLA	1.91	53.75	5.88
PLA GB 0.05	1.97	47.57	5.77
PLA GB 0.1	2.05	41.25	5.50
PLA GB 0.15	2.24	37.43	2.46

**Table 5:** Mean values of E_f_ and σ_f_ of pure PLA and composite 3D-printed samples—3-point bending tests.

Sample	E_f_ (GPa)	σ_f_ (MPa)
PLA	1.91	53.75
PLA GB 0.05	1.97	47.57
PLA GB 0.1	2.05	41.25
PLA GB 0.15	2.24	37.43

## Data Availability

Data are contained within the article.

## Abbreviations

WEEE	Waste Electric and Electronic Equipment
EU	European Union
PV	Photovoltaic
RM	Raw Materials
MEX	Material Extrusion
3D	Three Dimensional
CAD	Computer-aided Design
AM	Additive Manufacturing
PLA	Poly(Lactic) Acid
GB	Glass Beads
SEM	Scanning Electron Microscopy
DSC	Differential Scanning Calorimetry
EDX	Energy-dispersive X-ray Diffraction
ABS	Acrylonitrile butadiene styrene

## Appendix A

**Table A1:** Printer and filament settings.

Parameter (Printer Settings)	Value	Parameter (Filament Settings)	Value
Layer height (mm)	0.2	Diameter (mm)	1.75
No. of perimeters	2	Extrusion multiplier	1.1
Seam position	Rear	Bed temperature (°C)	60
Speed first layer (mm/s)	20	Nozzle first layer temperature (°C)	215
Speed others (mm/s)	40	Nozzle other layer temperature (°C)	210
Extruder width (mm)	0.45	Slow down if layer time is below	10 s

**Table A2:** Width (b) and thickness (d) of samples.

D638-10 TYPE IV ASTM (D) Samples (Neck)	b (mm)	d (mm)	ISO 178 (P) Samples	b (mm)	d (mm)
1D Pure PLA	6.02	3.56	1P Pure PLA	10.35	4.4
2D Pure PLA	6.17	3.61	2P Pure PLA	10.2	4.28
3D Pure PLA	5.98	3.39	3P Pure PLA	10.3	4.34
1D PLA_GB_0.05	6.26	3.67	1P PLA_GB_0.05	10.1	4.12
2D PLA_GB_0.05	6.16	3.61	2P PLA_GB_0.05	10.12	4.15
3D PLA_GB_0.05	6.14	3.61	3P PLA_GB_0.05	10.02	4.13
4D PLA_GB_0.1	6.1	3.49	4P PLA_GB_0.1	10.11	4.12
5D PLA_GB_0.1	6.09	3.63	5P PLA_GB_0.1	10.05	4.06
6D PLA_GB_0.1	6.21	3.63	6P PLA_GB_0.1	10.06	4.06
7D PLA_GB_0.15	6.03	3.54	7P PLA_GB_0.15	10.24	4.05
8D PLA_GB_0.15	6.29	3.45	8P PLA_GB_0.15	10.58	4.04
9D PLA_GB_0.15	6	3.34	9P PLA_GB_0.15	10.17	4.09
